# A case of naevus sebaceus of Jadassohn of the scalp in Jamaica

**DOI:** 10.1093/jscr/rjab552

**Published:** 2021-12-28

**Authors:** Geoffrey Williams, Gabriella Diaz, Garfield Blake

**Affiliations:** Division of Plastic & Reconstructive Surgery, Department of Surgery, Cornwall Regional Hospital, Montego Bay, Jamaica; Division of Plastic & Reconstructive Surgery, Department of Surgery, Cornwall Regional Hospital, Montego Bay, Jamaica; Department of Pathology, Cornwall Regional Hospital, Montego Bay, Jamaica

## Abstract

Naevus sebaceus (NS), also referred to as NS of Jadassohn, is a rare non-melanocytic congenital cutaneous hamartoma with mainly sebaceous differentiation. NS has pluripotent potential with the possible evolution of benign and/or malignant neoplastic transformation. Literature of clinical audit and retrospective analyses conclude that there is no need for prophylactic excision except for cases in which malignant transformation is suspected. Although malignant transformation is rare, there are psychosocial issues with which to contend. We present a case of a 5 year-old girl with a cerebriform mass to her right parietal scalp, which was present at birth.

## INTRODUCTION

Sebaceous glands occur over the entire body surface, with the exception of the volar aspect of the hands in addition to the dorsal and plantar aspects of the feet. The scalp possesses the largest number and most concentrated collection of these glands [1]. Numerous sebaceous glands surround each hair follicle in the scalp [2]. They produce sebum which lubricates the skin [1].

Naevus sebaceus (NS) was first described by Jadassohn in 1895; hence, it is known as NS of Jaddassohn [3]. NS is a non-melanocytic congenital cutaneous hamartoma with sebaceous differentiation, hyperplasia of apocrine glands, hair follicles and epidermis. It presents as a well-demarcated pink or skin-coloured verrucous plaque with focal alopecia [3–5]. The prevalence is 0.3% in neonates with an equal frequency in males and females of all races, and most commonly, it occurs as a solitary lesion with a predilection for the head and neck region at 95% [3, 4]. The diagnosis is usually straightforward on clinical grounds; however, histological examination is confirmatory [4].

There is a transition in tissue morphology; hormonal influences from the mother may briefly increase the prominence in an infant, whereas pubertal hormones enhance the verrucoid appearance in an adolescent [6]. Secondary neoplasms in NS can develop during adolescence and adulthood. The literature varies in the malignant transformation risk from 0.8–22% [3, 4]. Given the rarity of NS, it was challenging to find publications from the Caribbean; hence, the reason to contribute to the literature.

## CASE REPORT

The patient’s mother was gravida 3 and parity 3 who received antenatal care with the recommended antenatal ultrasounds which did not detect any congenital anomalies. There was no known family history of any mass lesions or other dermatological pathologies. She was born at term, with a mass to the right parietal region of the scalp, which was documented on her hospital chart. An magnetic resonance imaging brain study, done at 6 weeks of life, exhibited a ~3-cm diameter exophytic right parietal scalp mass that appeared to be superficial and separated from the cranium by a layer of scalp fat. There were no underlying osseous or brain abnormalities. The impression was that of a right parietal scalp mass with a benign appearance. Differential diagnoses included an epidermoid tumour or another benign tumour.

**Figure 1:**
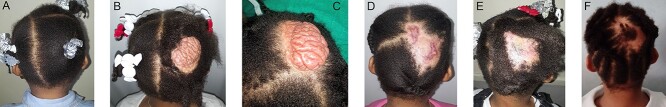
(**A**) NS is hidden by hair; (**B**) loosening of hair revealing NS; (**C**) NS prior to an excisional biopsy; (**D**) 57 days post-excision of and closure via a Rhombic flap; (**E**) 5 months post-excision; (**F**) 3 years post-excision.

The patient presented to the plastic surgery outpatient clinic at 5 years of age. The well-circumscribed pink cerebriform-like tumour was camouflaged by her hair styling (Fig. 1A). The measurements were 5 × 2 cm (Fig. 1B). The remainder of her examination was unremarkable and no dysmorphic features were noted. Her attainment of developmental milestones was normal and she was doing well at school.

One month after her first presentation to the clinic, an excisional biopsy of the mass was performed under general anaesthesia (Fig. 1C). The scalp defect was easily closed using a rhombic flap. A bolstered dressing was applied, and she was allowed to go home on that day. The specimen was marked and sent for histological examination. The patient was seen at the plastic surgery outpatient department weekly for the first 4 weeks, and she went on to complete healing without any eventualities. Thereafter, she was given 6-month appointments and subsequently yearly follow-ups (Fig. 1D–F).

The pathology report consisted of a 5 × 4 × 1.2 cm portion of cerebriform skin and 2.4 × 1.2 × 0.5 cm portion of grey brown tissue. The microscopic findings demonstrated skin, with the most prominent feature being the increase in the number of sebaceous glands. Many sebaceous glands communicate directly with the epidermis. The epidermis was hyperplastic in places. The overall features were those of NS. A separately submitted specimen was reported as a portion of loose fibrous tissue.

## DISCUSSION

The medical importance of a solitary NS relates to the possible evolution of benign or malignant neoplastic changes. NS has pluripotent potential [7]. A secondary tumour from a NS may differentiate into follicular, sebaceous, apocrine and eccrine cells and, rarely, a secondary tumour can differentiate into muscle [5, 7]. The literature varies in the malignant transformation risk from 0.8–22% [3] Old reports overestimate the frequency of malignant tumours due to a misdiagnosis of basal cell carcinoma (BCC) for trichoblastomas [6].

A 2014 retrospective analysis of 707 cases of NS revealed that trichoblastoma followed by syringocystadenoma papilliferum were the most frequent benign tumours arising from NS [8]. The malignant tumours comprised 2.5% of the specimens, with BCC as the most common [4, 8, 9] followed by squamous cell carcinoma. Almost all malignant tumours were seen in adults and the incidence of secondary neoplasms was statistically related to age and anatomic site (*P* < 0.05) [8]. An 18-year review suggested that the incidence was in the region of 0.8% [3].

A clinical audit published in 2007 concluded that prophylactic excision of NS is not warranted, especially in young children. Excision was recommended only when benign or malignant neoplasms are clinically suspected or for cosmetic reasons [10].

Although the more recent literature supports that malignant transformation may be less than previously documented, full excision was completed in this patient. In addition to the possible development of a secondary tumour, psychosocial problems, such as a high level of social anxiety, social avoidance, social discrimination and interaction, and interference with personal life must be taken into consideration. The dissatisfaction with facial appearance may lead to behavioural difficulties as well [11]. Three years post her excision, now at 8 years of age, there is much less alopecia and no new masses or cutaneous changes. She continues to do well in school, both academically and socially.

The rhombic flap was selected for scalp closure as it was easily designed, quick, provided excellent contour, texture, thickness and colour match and took advantage of the skin laxity adjacent to the defect [12, 13]. The rich anastomotic blood supply of the subdermal plexus provided the basis of circulation to this random-pattern flap without a requirement for the axiality of blood supply to be considered [13].

In conclusion, we would recommend that NS should be excised as early as possible to prevent the occurrence of any secondary neoplasms and to prevent psychosocial issues.

## CONFLICT OF INTEREST STATEMENT

None declared.

## FUNDING

None.
